# A nested case-control study of 277 prediagnostic serum cytokines and glioma

**DOI:** 10.1371/journal.pone.0178705

**Published:** 2017-06-08

**Authors:** Judith Schwartzbaum, Min Wang, Elisabeth Root, Maciej Pietrzak, Grzegorz A. Rempala, Ruo-Pan Huang, Tom Borge Johannesen, Tom K. Grimsrud

**Affiliations:** 1 Division of Epidemiology, College of Public Health, Ohio State University, Columbus, Ohio, United States of America; 2 Comprehensive Cancer Center, Ohio State University, Columbus, Ohio, United States of America; 3 Mathematical Biosciences Institute, Ohio State University, Columbus, Ohio, United States of America; 4 Department of Biomedical Informatics, Ohio State University, Columbus, Ohio, United States of America; 5 Department of Geography, Ohio State University, Columbus, Ohio, United States of America; 6 Division of Biostatistics, College of Public Health, Ohio State University, Columbus, Ohio, United States of America; 7 RayBiotech, Inc., Norcross, Georgia, United States of America; 8 RayBiotech, Inc. Guangzhou, China; 9 Department of Registration, Cancer Registry of Norway, Oslo, Norway; 10 Department of Research, Cancer Registry of Norway, Oslo, Norway; University of Alabama at Birmingham, UNITED STATES

## Abstract

Recent research shows bidirectional communication between the normal brain and the peripheral immune system. Glioma is a primary brain tumor characterized by systemic immunosuppression. To better understand gliomagenesis, we evaluated associations between 277 prediagnostic serum cytokines and glioma. We used glioma (n = 487) and matched control (n = 487) specimens from the Janus Serum Bank Cohort in Oslo, Norway. Conditional logistic regression allowed us to identify those cytokines that were individually associated with glioma. Next, we used heat maps to compare case to control Pearson correlation matrices of 12 cytokines modeled in an *in silico* study of the interaction between the microenvironment and the tumor. We did the same for case-control correlation matrices of lasso-selected cytokines and all 277 cytokines in the data set. Cytokines related to glioma risk (*P* ≤ .05) more than 10 years before diagnosis are sIL10RB, VEGF, beta-Catenin and CCL22. LIF was associated with decreased glioma risk within five years before glioma diagnosis (odds ratio (OR) = 0.47, 95% confidence interval (CI) = 0.23, 0.94). After adjustment for cytokines above, the previously observed interaction between IL4 and sIL4RA persisted (> 20 years before diagnosis, OR = 1.72, 95% CI = 1.20, 2.47). In addition, during this period, case correlations among 12 cytokines were weaker than were those among controls. This pattern was also observed among 30 lasso- selected cytokines and all 277 cytokines. We identified four cytokines and one interaction term that were independently related to glioma risk. We have documented prediagnostic changes in serum cytokine levels that may reflect the presence of a preclinical tumor.

## Introduction

Advances in immunology have led to development of promising therapies for treatment of glioma [1, 2]. However, at present, the median survival time from diagnosis of the most common adult type of glioma, glioblastoma, is only 14 months [3]. This brief survival time may be attributable to the fact that known preclinical symptoms occur, on average, only three months before diagnosis[4] and often indicate an advanced tumor. However, glioma development prior to the appearance of symptoms may be a longer process [5]. It is therefore important that the early stages of gliomagenesis be identified and examined with the ultimate goal of intervention.

Recent research has established two-way communication between the central nervous system and the immune system in the absence of pathology in either [6]. Consistent with these findings, epidemiological, biological and clinical evidence support the role of the immune system in gliomagenesis. For example, elevated levels of prediagnostic total serum IgE are associated with reduced glioma risk [7], as is a history of varicella zoster infection [8]. The tumor interacts with microglia in its microenvironment to escape immune surveillance [9, 10]. Furthermore, glioma patients may exhibit severe systemic immune suppression [11]. Thus we investigated prediagnostic cytokines, indicators of immune function, to determine whether they were related to subsequent glioma risk or indicate the presence of a preclinical tumor.

Cytokines are multi-functional signaling proteins that, among other roles, regulate immune responses including tumor immune surveillance, tumor-induced immunosuppression [12] and also promote neural stem cell renewal and astrocyte differentiation [13]. In the glioma microenvironment they are eventually appropriated by the tumor and assist in immune suppression and tumor invasion [9, 14]. Because of their obvious relevance to gliomagenesis there are several studies of the relationship between cytokines and glioma after diagnosis [15–17], however, our previous study of 12 allergy and glioma related serum cytokines is the only known examination of prediagnostic serum cytokines and glioma [18]. In that study we found an interaction between interleukin 4 (IL4), a cytokine active in allergy [19] and normal brain function [20]), and its soluble receptor, interleukin 4 receptor alpha (sIL4RA). One goal of the present study was to determine whether we would identify additional associations between individual prediagnostic serum cytokines and subsequent diagnosis of glioma and, if so, whether the previously observed interaction between IL4 and sIL4RA [18] would persist in their presence. A difference between our previous and present study is that, because of small numbers of glioblastoma cases in the five years before diagnosis (n = 22), we did not analyze the data separately by this subgroup in the present study.

Cytokines are difficult to study individually because they are pleiotropic, redundant, have paradoxical functions and operate in complex non-linear networks where they may activate or inhibit each other [21]. In our previous study of a subset of 12 allergy-related cytokines [18] from the present data set, we found that correlations among case but not control cytokines weaken within the five years prior to glioma diagnosis. To attempt replication of these findings using different sets of cytokines, we estimated case-control correlations among 12 of 15 cytokines included in an *in silico* study of prediagnostic cytokines in the glioma microenvironment [14]. In this study, Wu et al. found that, as the tumor took control of cytokine production, the previously strong correlations among cytokines in the microenvironment diminished. Whether we would see a corresponding cytokine correlation diminution in the peripheral circulation, prior to the time of diagnosis, was one of the questions that we addressed. In addition to the 12 cytokines identified by Wu et al, we examined case-control correlations among cytokines selected by the lasso method of variable selection [22] and all 277 prediagnostic cytokines in the data set.

Our sample is an individually matched, nested case-control study of 277 cytokines based on prospectively collected sera from 487 subsequent glioma cases and their 487 matched glioma controls from the Janus Serum Bank in Oslo, Norway.

## Methods

### Study population

The study population has previously been described in detail [18]. Here we briefly note its salient features. The Janus Serum Bank was established in 1972 to conduct epidemiological studies of cancer [23, 24]. This biobank is now owned by the Cancer Registry of Norway and contains serum samples from 166,137 men and 152,491 women. Approximately 90% of the serum donors were participants in routine cardiovascular health examinations conducted by the National Health Screening Services. Samples were stored at −25°C and underwent one thaw–freeze cycle in preparation for the present study.

The final data set contained no personal identifiers. However, initially, personal identification numbers were used to link Janus Serum Bank cohort members to the Cancer Registry of Norway. We analyzed serum samples from 487 blood donors who were subsequently diagnosed with glioma (International Classification of Disease, Oncology, Third Edition [ICD-O-3] morphology codes 9380–9411, 9420–9480, and 9505) between January 1, 1974 and December 31, 2007.

A control participant for each glioma case was randomly selected, according to an incidence density sampling scheme, from the same cohort. Controls were individually matched to cases on date of blood collection (±3 months), date of birth (±1.25 years), county of residence at blood collection and gender. Matched control participants were required to be alive on the date of diagnosis of the case to which they were matched and free from any cancer except non-melanoma skin cancer. In addition, people diagnosed with rare tumors (*i*.*e*., all tumors other than breast, prostate, and colorectal) after the corresponding case’s date of glioma diagnosis were not included. We analyzed serum samples from 487 control participants.

### Ethics statement

The research plan on which the present study is based was approved by the Regional Ethics Committee of Southern Norway and the Norwegian Data Protection Authority. During the Janus Serum Bank’s first years, 1973–1992, donors gave broad verbal consent for use of samples in “cancer research” [25] in accordance with Norwegian law which did not require written consent at that time. No samples were collected from 1993 to 1996. Samples from 1997 and later were collected in conjunction with an explicit written informed consent document (Act Relating to Biobanks, § 12, http://ec.europa.eu/research/biosociety/pdf/norwegian_act_biobanks.pdf). These signed forms are stored either at the Cancer Registry of Norway or the Norwegian Institute of Public Health. The Norwegian Data Protection Authority (https://www.datatilsynet.no/English/) has approved of the use of the Janus data and biological samples collected during the period 1972–2004, while requiring that blood donors are free to unconditionally withdraw their consent at any time. Upon withdrawal, their serum samples will be destroyed and associated data deleted (Act Relating to Biobanks, § 14, http://ec.europa.eu/research/biosociety/pdf/norwegian_act_biobanks.pdf). As additional participant protection all research projects using specimens from the Janus repository and data from the Cancer Registry of Norway need approval from a Regional Committee for Medical and Health Research Ethics. Donors are informed about ongoing research projects through the Cancer Registry web pages (http://www.kreftregisteret.no/en/Research/About-our-Research/). Analyses of these anonymous data were also approved by the Institutional Review Board of Ohio State University.

### Cytokine microarray analysis: RayBio^®^ human cytokine antibody array kits

Analytic methods are discussed in detail elsewhere [18], here we briefly summarize these methods. Cytokine array kits, consisting of a combination of two Human Cytokine Antibody Arrays (G2000, n = 174 and G4000, n = 274) from RayBiotech, Inc. (Norcross, Georgia) were used to measure 277 serum cytokines, soluble cytokine receptors and transcription factors. These array kits were mailed to Professor Eivind Hovig's Laboratory at Oslo University, Norway where serum samples were randomly assigned to print batches. Laboratory personnel did not know the case status of the study participants from whom the samples were drawn. The antibody- based microarray assay is analogous to a sandwich ELISA assay using two sets of anti-cytokine or transcription factor antibodies.

### Statistical methods

This article is the second analyzing prediagnostic serum cytokine levels as they affect glioma risk and are affected by the preclinical tumor. The first manuscript [18] was based on the *a-priori* hypothesis of an association between allergy-related cytokines and glioma and was restricted to 12 of the 277 cytokines in the data set. In the present manuscript we examine all 277 of these cytokines and two cytokine subsets that differ from those in the previous study. We stratify our findings by time before diagnosis (≤ 5 years, > 10 years, >15 years, >20 years, all times).

We first minimized the potential influence of outliers by transforming serum cytokine values to a natural logarithmic scale and then standardizing each to a mean of zero and a standard deviation of one. To estimate odds ratios for each of the 277 cytokines, we used conditional logistic regression models, conditioned on matched case-control sets and stratified by the prediagnostic time categories. This method yielded seven statistically significant (*P* ≤ .05) cytokines individually associated with glioma. Cytokine levels are highly correlated with each other; we therefore used stepwise conditional logistic regression (backward selection) to identify the cytokines, among those individually related to glioma, which continued to be associated with glioma when all seven cytokines were included in the same regression model. When the models for each time category were selected, we included IL4, sIL4RA and their interaction term in these models to see if these variables, identified in our previous study [18], retained their significance in the presence of the newly identified cytokines. These three IL4 terms were retained in the models if the interaction term was statistically significant. Cytokines which lost their significance in the presence of these IL4 terms were also removed from the models. In addition, to identify patterns in the relative sizes of the odds ratios by time before diagnosis, we ordered each of the 277 individual odds ratios by magnitude and time category whether or not they were statistically significant.

To understand the inter-relationships among cytokines reported in a previous *in silico* study of the glioma microenvironment [14], we generated a network that included seven of the 15 cytokines in that study (IPA®, QIAGEN Redwood City, www.qiagen.com/ingenuity).

To compare case to control correlation coefficients of 12 of the 15 *in silico* cytokines on which we had data, we used heat maps stratified by time before diagnosis. Next, to identify additional cytokines that discriminated between cases and controls in our sample, we used the lasso method of variable selection [22]. We again used heat maps to investigate correlations among these lasso-selected variables and all 277 cytokines. These heat maps were based on a hierarchical clustering method with a distance metric computed from the Pearson correlations. For comparison of the heat maps across time periods, cytokines were aligned in the same order for each time period.

To further quantify our visual interpretations of the heat maps containing correlations among 277 cytokines, we created 1000 bootstrap samples (sampled with replacement) stratified by time before diagnosis. We then summed the absolute values of case or control correlation coefficients separately. Next, for each time category, we subtracted the case sums from the control sums and calculated the mean differences and 95% confidence intervals of the bootstrap samples. A positive mean value would indicate that, on average, control correlations were larger than were those of cases. A negative value would indicate the opposite.

To evaluate quality control, for each of the cytokines that were identified by the logistic regression models above or were included in heat maps based on the *in silico* study [14], we used the 47 replicate case and 48 replicate control samples to estimate coefficients of variation (CV) for each cytokine, and then calculated their median values as well as their interquartile ranges (IQR).

All analyses were conducted using SAS statistical software, version 9.3 (SAS Institute Inc, Cary, NC) or the R language and environment (R Core Team (2013). R: A language and environment for statistical computing. R Foundation for Statistical Computing, Vienna, Austria. (URL http://www.R-project.org/)).

## Results

### Characteristics of study population

Cases and controls are balanced with respect to the percentage of men, median age at blood collection and year of glioma diagnosis (Table 1). However, because age 42 years was the median age at blood collection, with little variation (IQR = 40, 43), there is a 22 year age difference between the median age at diagnosis among study participants whose blood was collected within five years before glioma diagnosis and those whose blood was collected more than 20 years before glioma diagnosis. In the Sl Table, descriptive variables are shown separately for glioblastoma (grade 4) and other glioma (grades 1–3). Findings are similar to those in Table 1 however, in the S1 Table there are large and consistent differences in the percentage of men between the two tumor types. Overall, there is a larger percentage of men with glioblastoma, (median = 72 percent, IQR = 67, 77 percent) than with other types of glioma, (median = 58 percent, IQR = 51, 66 percent).

**Table 1:** Descriptive characteristics of sample^1^ by time of blood sample before case diagnosis.

Descriptive variable	Glioma	Controls^2^
**Total**		
Number	487	487
Percent men (mean)	67 (63, 71)^3^	67 (63, 71)
Median age at blood collection	42 (40, 43)^4^	42 (40, 43)
Median year of blood collection	1986 (1976, 1989)	1986 (1976, 1989)
Median age at glioma diagnosis	57 (51, 63)	----^5^
Median years from blood collection to diagnosis	15 (9, 21)	----
**≤ 5 years before diagnosis**		
Number	55	55
Percent men (mean)	58 (45, 72)	58 (45, 72)
Median age at blood collection	42 (41, 46)	42 (41, 47)
Median year of blood collection	1988 (1984, 1989)	1988 (1984, 1989)
Median age at glioma diagnosis	45 (43, 48)	----
Median years from blood collection to diagnosis	3 (1, 4)	----
**> 10 years before diagnosis**		
Number	347	347
Percent men (mean)	67 (62, 72)	67 (62, 72)
Median age at blood collection	42 (41, 46)	42 (41, 46)
Median year of blood collection	1985 (1975, 1989)	1985 (1975, 1989)
Median age at glioma diagnosis	59 (55, 66)	----
Median years from blood collection to diagnosis	17 (14, 24)	----
**> 15 years before diagnosis**		
Number	228^6^	230
Percent men (mean)	65 (59, 71)	65 (59, 71)
Median age at blood collection	42 (40, 44)	42 (40, 43)
Median year of blood collection	1977 (1975, 1987)	1977 (1975, 1988)
Median age at glioma diagnosis	63 (59, 68)	----
Median years from blood collection to diagnosis	21 (17, 27)	----
**> 20 years before diagnosis**		
Number	126	126
Percent men (mean)	67 (59, 76)	67 (59, 76)
Median age at blood collection	41 (38, 44)	41 (38, 44)
Median year of blood collection	1976 (1973, 1977)	1976 (1973, 1977)
Median age at glioma diagnosis	67 (63, 71)	----
Median years from blood collection to diagnosis	26 (23, 30)	----

1 Glioma study participants were blood donors (1974–2007) to the Janus Serum Bank, Oslo, Norway.

2 Control participants were individually matched to cases on age date of blood collection and sex.

3 95% confidence interval

4 Interquartile range

5 Not applicable

6 Controls are matched to cases within three months of blood collection. Therefore a matched pair may fall into separate time categories thus accounting for unequal numbers of cases and controls in this category.

### Case-control cytokine level means and associated odds ratios

Table 2 shows means of case and control standardized logs of cytokine levels by time before diagnosis and their associated statistically significant (*P* ≤ .05) odds ratios. The directions of differences between case and control cytokine level means are consistent the magnitudes of the related odds ratios. Odds ratios for soluble interleukin 10 receptor beta (sIL10RB) are similar among people whose blood was drawn more than ten years before diagnosis, while those for the IL4-sIL4RA interaction increase slightly with the length of time before diagnosis. The inverse association between leukemia inhibitory factor (LIF) and glioma is restricted to participants whose blood was drawn five years before glioma diagnosis. Complete results of the analyses of all 277 cytokines by the five time categories are included in the S2 Table.

**Table 2:** Mean case-control levels of selected^1^ cytokines^2^ and their associations with glioma by time before diagnosis.

Cytokines	Glioma Mean^3^ (95% CI)	Control Mean (95% CI)	Odds Ratio^4^	95% CI^5^
	**Total**			
	**487 cases/487 controls**^6^			
**sIL10RB**	-0.05 (-0.13, 0.04)	0.04 (-0.05, 0.13)	0.69	0.55,0.87
**VEGF**	0.05 (-0.04, 0.14)	-0.05 (-0.14, 0.04)	1.46	1.18,1.82
**IL4**	0.01 (-0.08, 0.10)	-0.01 (-0.10, 0.08)	1.13	0.90,1.43
**sIL4RA**	-0.01 (-0.10, 0.08)	0.03 (-0.06, 0.11)	0.92	0.76,1.12
**IL4-sIL4RA**	-0.05 (-0.15, 0.16)	-0.24 (-0.34, -0.15)	1.37	1.16,1.61
	**≤ 5 Years before Diagnosis**			
	**55 cases/55 controls**			
**LIF**	-0.23 (-0.52, 0.05)	0.05 (-0.21, 0.30)	0.47	0.23, 0.94
	**> 10 Years before Diagnosis**			
	**347 cases/347 controls**			
**sIL10RB**	-0.02 (-0.13, 0.08)	0.11 (0.00, 0.22)	0.56	0.42, 0.75
**VEGF**	0.10 (-0.01, 0.21)	-0.03 (-0.13, 0.07)	1.58	1.22,2.05
**IL4**	0.05 (-0.06, 0.16)	0.01 (-0.10, 0.11)	1.35	1.01, 1.79
**sIL4RA**	-0.09 (-0.20, 0.02)	0.00 (-0.10, 0.10)	0.84	0.67,1.07
**IL4-sIL4RA**	-0.06 (-0.18, 0.07)	-0.25 (-0.35, -0.15)	1.42	1.15, 1.74
	**> 15 Years before Diagnosis**			
	**228 cases/230**^7^ **controls**			
**sIL10RB**	0.06 (-0.07, 0.19)	0.24 (0.10, 0.38)	0.51	0.36, 0.71
**beta-Catenin**	0.16 (0.02, 0.30)	0.01 (-0.12, 0.13)	1.86	1.28, 2.71
**CCL22**	0.06 (-0.07, 0.19)	-0.11 (-0.25, 0.03)	1.45	1.07, 1.96
**IL4**	0.13 (-0.01, 0.27)	0.11 (-0.03, 0.24)	1.03	0.71,1.50
**sIL4RA**	-0.15 (-0.29, -0.01)	-0.07 (-0.19, 0.05)	0.72	0.54, 0.97
**IL4-sIL4RA**	-0.02 (-0.20, 0.16)	-0.26 (-0.40, -0.13)	1.58	1.22,2.04
	**> 20 Years before Diagnosis**			
	**126 cases/126 controls**			
**sIL10RB**	0.34 (0.16, 0.52)	0.49 (0.30, 0.68)	0.53	0.33, 0.84
**CCL22**	0.01(-0.17, 0.20)	-0.22 (-0.41, -0.02)	1.53^8^	1.03, 1.26
**IL4**	0.35 (0.15, 0.55)	0.29 (0.12, 0.47)	1.28	0.69, 2.39
**sIL4RA**	-0.38 (-0.57, -0.19)	-0.42 (-0.58, -0.26)	0.83	0.56,1.22
**IL4-sIL4RA**	0.04 (-0.25, 0.33)	-0.37 (-0.58, -0.17)	1.72	1.20, 2.47

1. All 277 cytokines were tested and seven individually statistically significant (*P*≤. 05) cytokines were included in stepwise regression models. Five cytokines in table retained statistical significance in stepwise models. The IL4 interaction term was added to the models and retained if significant.

2. Abbreviations: sIL10RB, soluble interleukin 10 receptor beta; VEGF, vascular endothelial growth factor; IL4, interleukin 4; sIL4RA, soluble interleukin 4 receptor alpha, IL4-sIL4RA, interaction between IL4 and sIL4RA; LIF, leukemia inhibitory factor; beta-Catenin, Catenin beta-1; CCL22, C-C motif chemokine 22

3. Mean values are means of standardized natural logarithms of the cytokine levels.

4. Logistic regression conditioned on matched set (age, date of blood collection, sex), adjusted for other cytokines in table

5. 95% confidence interval

6. Controls are assigned the date of diagnosis of the case to which they were matched.

7. Controls are matched to cases within three months of blood collection. Therefore a matched pair may fall into separate time categories thus accounting for unequal numbers of cases and controls in this category

8. The odds ratio for CCL22 of 1.53 among people whose blood was drawn more than 20 years before glioma diagnosis shows that a one unit increase in the standardized log of cytokine levels is associated with a 53% increase in the odds of glioma.

### Network of previously identified cytokines associated with glioma

**Fig 1:**
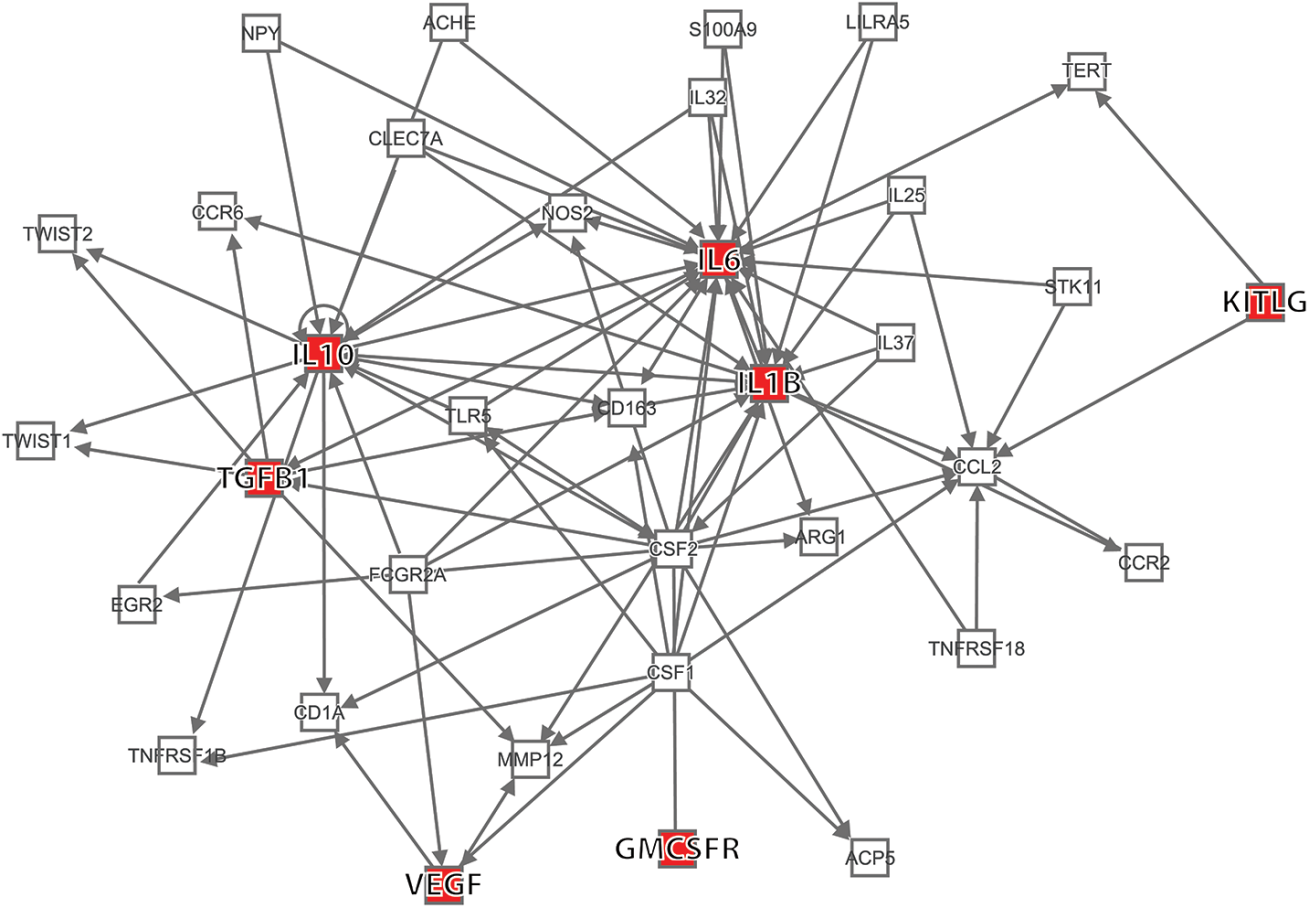
Network includes seven of 15 previously cytokines identified by Wu et al. [14]. Abbreviations: IL6, interleukin 6; IL10, interleukin 10; IL1B, interleukin 1beta;KITLG, stem cell factor, kit ligand; TGFB1, transforming growth factor beta1; VEGF, vascular endothelial growth factor; GMCSFR, Granulocyte macrophage colony stimulating factor receptor.

Fig 1 shows the known networks among seven of the 15 cytokines included Wu et al’s *in silico* study [14]. These cytokines were used in their models to study the prediagnostic interaction between the microenvironment and glioma. Rather than studying associations between these cytokines and glioma one at a time, we compared case and control correlations among them. Our rationale for this approach was that interrelationships among these cytokines may distort estimates of effects of individual cytokines on glioma risk.

### Correlations among 12 previously identified case and control cytokines

**Fig 2:**
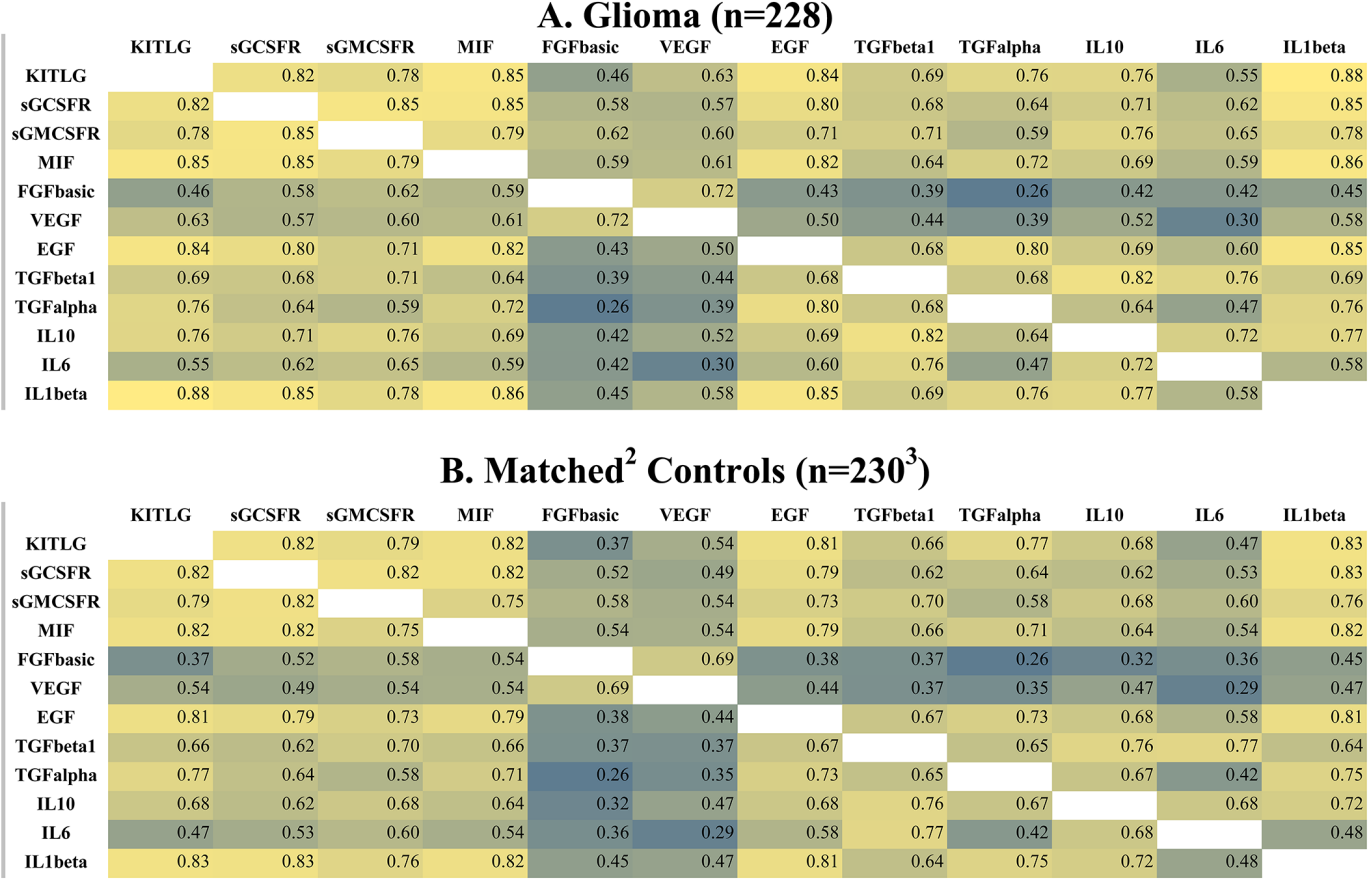
Correlations among cytokines > 15 years before glioma diagnosis. Color scale: yellow-highest correlations, green = moderate correlations, blue = lowest correlations; Abbreviations: KITLG, stem cell factor, kit ligand; sGCSFR, soluble granulocyte colony stimulating factor receptor; sGMCSFR, soluble granulocyte macrophage colony stimulating factor receptor; MIF, macrophage migration inhibitory factor; FGFbasic, basic fibroblast growth factor; VEGF, vascular endothelial growth factor; EGF, epidermal growth factor; TGFbeta1, transforming growth factor beta1; TGFalpha1, transforming growth factor alpha1; IL10, interleukin 10; IL6, interleukin 6; IL1beta, interleukin 1 beta.

Fig 2 displays case (A) and control (B) cytokine correlation patterns among 12 cytokines of the 15 identified in Wu et al’s *in silico* study [14]. In this study they found that, as the tumor entered a rapid growth phase, correlations among cytokines in the microenvironment rapidly diminished. The heat maps in Fig 2 are based on analysis of data from serum samples collected more than 15 years before glioma diagnosis. Correlation patterns among cases (A) and controls (B) are similar.

**Fig 3:**
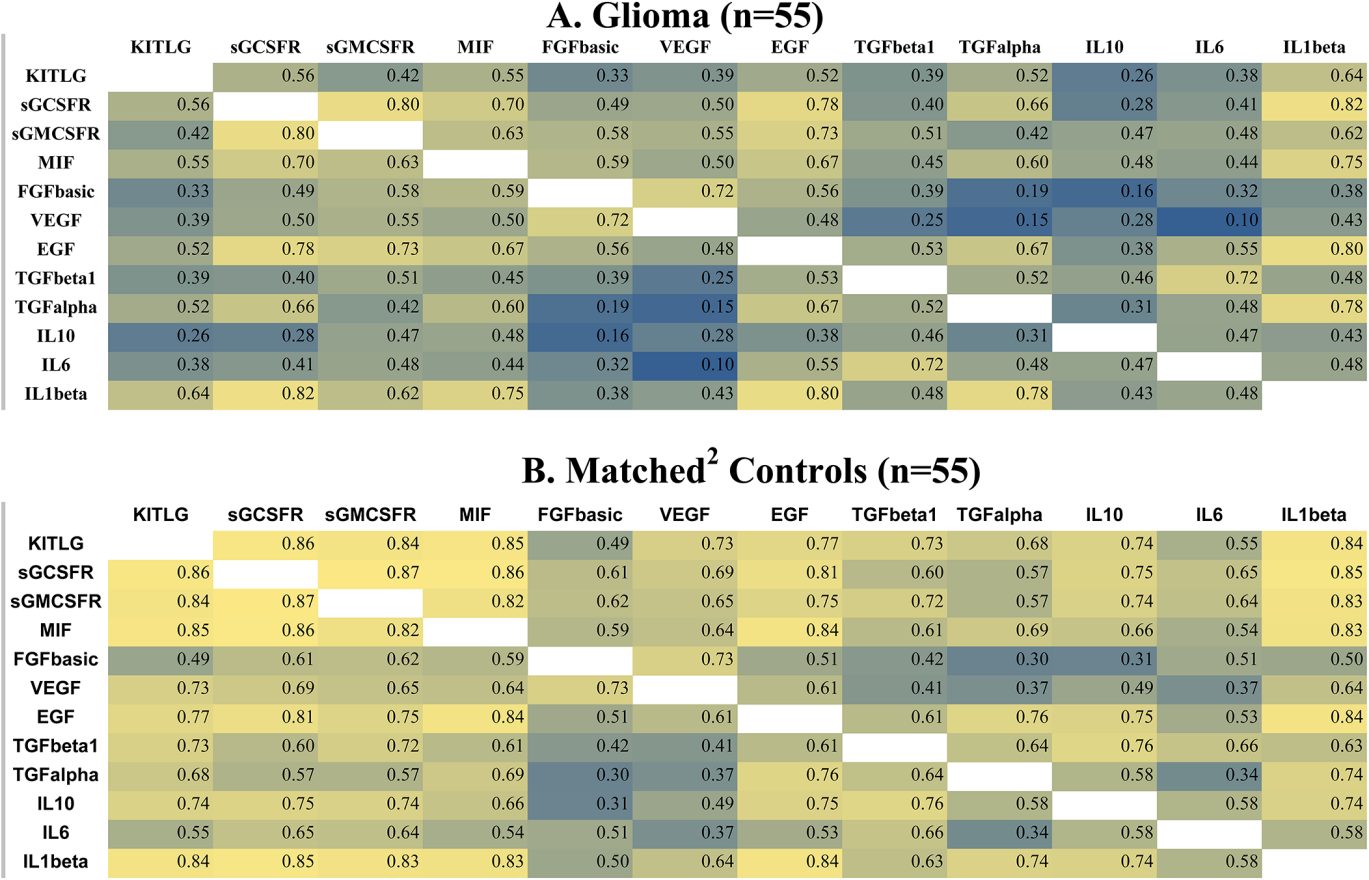
Correlations among cytokines ≤ 5 years before glioma diagnosis. Color scale: yellow-highest correlations, green = moderate correlations, blue = lowest correlations; Abbreviations: KITLG, stem cell factor, kit ligand; sGCSFR, soluble granulocyte colony stimulating factor receptor; sGMCSFR, soluble granulocyte macrophage colony stimulating factor receptor; MIF, macrophage migration inhibitory factor; FGFbasic, basic fibroblast growth factor; VEGF, vascular endothelial growth factor; EGF, epidermal growth factor; TGFbeta1, transforming growth factor beta1; TGFalpha1, transforming growth factor alpha1; IL10, interleukin 10; IL6, interleukin 6; IL1beta, interleukin 1 beta.

In Fig 3 case (A) and control (B) correlation patterns are based on serum collected within five years before diagnosis. Case correlation patterns (A) are clearly weaker (closer to the null) overall than are those among controls (B). This pattern is consistent with that found by Wu et al [14]. Furthermore, control patterns in Fig 3 are similar to those in Fig 2 suggesting that case-control differences in Fig 3 are not exclusively attributable to sampling variation resulting from the relatively small number of observations (n = 55 each for cases and controls).

In the S1 Fig case-control distributions of *P*-values, based on t-tests of the correlation coefficients in Fig 3, reveal patterns similar to those in Fig 3. Further stratifying on tumor grade (glioblastoma and other glioma) again confirms stronger correlations among controls (S2B andS2D Fig)) than cases (S2A and S2C Fig, although the case-control contrast is stronger for other glioma (grades 1–3) than for glioblastoma (grade 4).

### Mean serum cytokine levels of 12 previously identified cytokines ≤5 and >15 years before glioma diagnosis

Within five years before diagnosis, because there are weaker correlations among case than among control cytokines (Fig 3), one might expect mean case serum cytokine levels to be lower than are those of controls. As shown in the S3 Table, within five years before diagnosis, the standardized logs of 10 of 12 case serum cytokine levels are lower than are those of controls. In the S4 Table, more than 15 years before diagnosis, only eight of the 12 case cytokine levels are lower than are those of controls. In addition, differences between case and control cytokine levels are smaller in the S4 Table (>15 years) than they are in the S3 Table (≤5 years).

### Correlations among 30 lasso selected and all 277 cytokines ≤ 5 and >10 years before glioma diagnosis by case status

**Fig 4:**
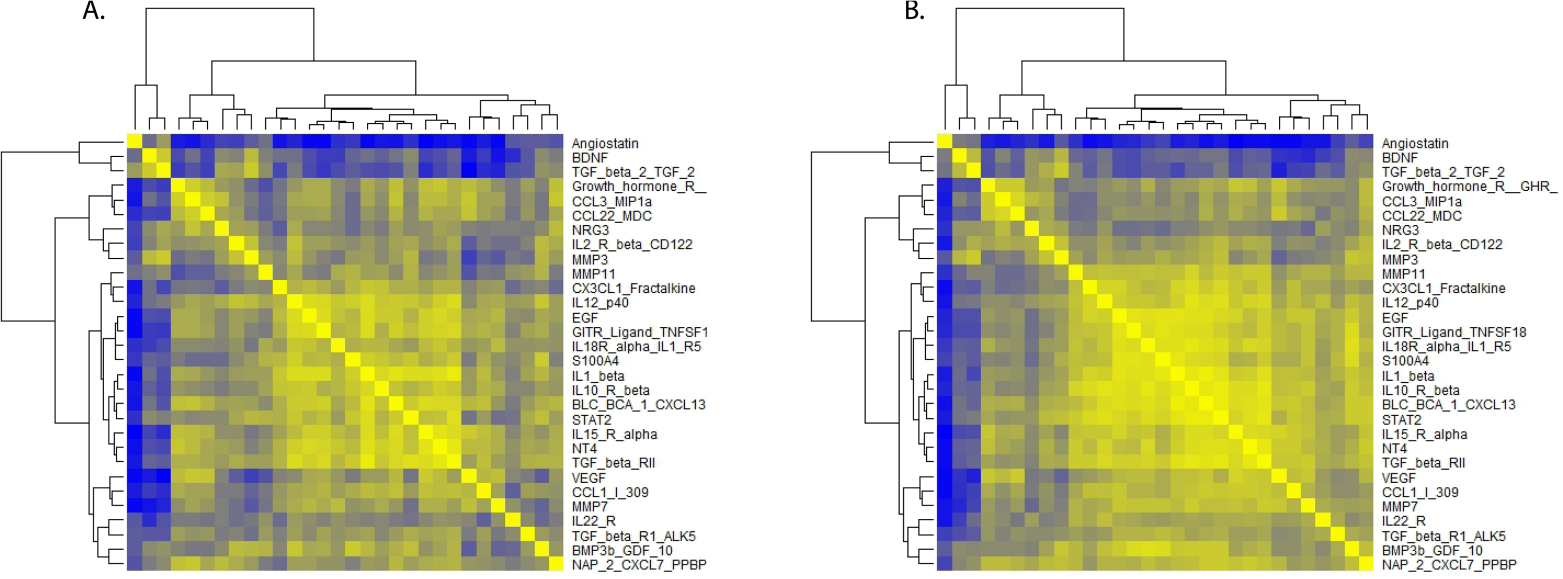
Correlations among cytokines ≤ 5 years before glioma diagnosis. Color scale: yellow-highest correlations, green = moderate correlations, blue = lowest correlations. For nomenclature see https://www.raybiotech.com/cytokine-nomenclature.html.

Within five years before diagnosis, correlation patterns among the 30 cytokines selected by the lasso variable selection method (Fig 4) are stronger among controls (B) than among cases (A). This pattern is repeated when correlations among all 277 cytokines are considered (S3 Fig). The top two heat maps contain correlations among cases (S3A Fig) and controls (S3B Fig) whose blood was collected within five years of diagnosis. The bottom two heat maps show that case (S3C Fig) and control (S3D Fig) correlations more than ten years before diagnosis are similar to each other.

### Relative magnitudes of odds ratios ≤ 5 years before diagnosis

These case-control correlation differences are also reflected in the relative magnitudes of the odds ratios by time category (S2 Table). When the 277 odds ratios in the five time categories (≤ 5 years, > 10 years, >15 years, >20 years, all times) are ordered by size, 90 out of the 100 lowest odds ratios in the data set are found among people whose blood was collected within five years before diagnosis. Only 13 of the 100 highest odds ratios in the data set are found within the lowest time category.

### Confirm visual impressions of heat maps using bootstrap analysis

To further quantify our visual impressions of the heat maps, within the five time categories, we created 1000 bootstrap samples and calculated the differences between the sums of the absolute values of case from control correlations by time before diagnosis. We show the mean values of these differences by time category in Table 3. A positive value for the difference indicates control correlations are larger than case correlations and a negative value suggests the opposite. Therefore within five years before diagnosis, control correlations are stronger than are those among cases (mean difference = 3088.06; 95% CI = -1823.47, 7907.50), although these results are not statistically significant (that is, the 95% confidence interval includes zero). Nonetheless, the direction of this difference is consistent with our visual impressions of these heat maps.

**Table 3:** Mean differences between case and control sums of absolute values of correlation coefficients of 277 cytokines in 1000 bootstrap samples by time before diagnosis.

**All study participants, n (cases/controls)**	487/487
Mean difference between sums (95% CI^1^)	-488.29^2^ (-2276.27, 1253.00)^3^
**Time from blood collection to tumor diagnosis**^4^	
**≤ 5 years before diagnosis, n**	55/55
Mean difference between sums (95% CI)	3088.06^5^ (-1823.47, 7907.50)
**>10 years before diagnosis, n**	347/347
Mean difference between sums (95% CI)	-1253.99 (-3436.83, 728.58)
**>15 years before diagnosis, n**	228/230^6^
Mean difference between sums (95% CI)	-1254.36 (-3963.36, 1391.64)
**>20 years before diagnosis, n**	126/126
Mean difference between sums (95% CI)	-1258.86 (-4589.04, 2001.73)

1 95% confidence interval

2 Negative differences mean that, on average, control correlations coefficients are larger than are those of cases.

3 If the 95% confidence interval includes zero then its corresponding *P*-value is not statistically significant (*P* < .05)

4 Controls were assigned the date of diagnosis of the case to which they were matched

5 Positive difference means that, on average, case correlation coefficients are larger than are those of controls.

6 Controls are matched to cases within three months of the time of blood collection. Therefore a matched pair may fall into separate time categories thus accounting for unequal numbers in this time category.

### Quality control: Coefficients of variation

The median coefficients of variation (CVs) and interquartile ranges (IQR) for the 16 cytokines included in Table 2 or Fig 2 are shown in the S5 Table. Case and control CV medians are each 0.07. All coefficients of variation are less than 0.11 except for those for vascular endothelial growth factor (VEGF) and beta-Catenin. In a previous study of 12 allergy-related cytokines using this data set [18], we found that batches were evenly distributed among cases and controls, as are rescanned samples.

## Discussion

We found three positive (VEGF, beta-Catenin and C-C motif chemokine 22 (CCL22)) and two negative (LIF, sIL10RB) associations between five prediagnostic serum cytokines and the subsequent risk of glioma. The previously noted interaction between IL4 and sIL4RA [18] continued to be associated with glioma in the presence of our univariate findings. We also confirmed earlier findings [18] of weaker correlations among case than among control cytokines within five years before glioma diagnosis. We observed this pattern among 12 cytokines independently identified in the previous literature [14], 30 cytokines selected by the lasso method of variable selection [22] and all 277 cytokines in the data set.

Our results indicating that VEGF, beta-Catenin and CCL22 levels increase glioma risk are consistent with previous post diagnostic and experimental literature [10, 16, 17, 26–28]. The association between VEGF, an angiogenic growth factor, and glioma is well-documented [16, 17, 26]. Beta-Catenin facilitates growth of brain tumor initiating cells [28] and CCL22, secreted by the tumor, recruits immunosuppressive regulatory T cells [10, 27]. Given these functions, positive associations with glioma risk are not unexpected.

Our observation of decreased glioma risk with higher levels of LIF was unexpected because this cytokine is a mediator of glioblastoma stem cell renewal [29] and a post- diagnostic case-control study of serum cytokines found higher levels of this cytokine among glioma cases than among controls [17]. However, LIF is a pleiotropic cytokine that also promotes glial cell differentiation [30, 31] and, under certain conditions, inhibits astrocyte proliferation [31]. These latter functions are consistent with our findings.

Similarly, we observed that elevated sIL10RB levels appear to reduce glioma risk. Previous literature characterizes IL10 as immunosuppressive [16] and finds only positive associations between serum IL10 [17] or genetic expression of IL10RB [15] and glioma. However, IL10 may also stimulate the immune system thereby playing a role in tumor immune surveillance [32]. IL10 knockout mice showed weakened tumor immune surveillance [33]. In addition, a soluble cytokine receptor (a receptor found in the serum rather than on the cell surface, e.g. sIL10RB) may act either as an antagonist or agonist [34].

Expression of IL4, an immunomodulatory cytokine [35], is increased in a glioblastoma cell line [16] where it is thought to be a component of tumor-induced immunosuppression [10]. However, the soluble receptor, sIL4RA, modulates IL4 [35] so the mechanism by which this interaction would increase glioma risk more than 20 years before diagnosis is not apparent.

Regardless of the consistency of our individual statistically significant findings with the post diagnostic literature, we suggest caution in their interpretation. We identified the five univariate cytokine associations by conducting 1,385 tests of statistical significance (277 multiplied by five time categories). Overall, only seven odds ratios were significantly associated with glioma (0.5%). (Two cytokines were eliminated using stepwise regression). This percentage is well within the false positive range (5%) and therefore, in addition to their consistency with previous literature, as noted above, these odds ratios findings must be replicated in another data set to achieve credibility. Furthermore, as we noted in the introduction, cytokines are signaling proteins that interact with each other so that individual serum cytokine levels may not be biologically meaningful [14]. Therefore, we suggest that our cytokine interaction term and correlation results better represent the known biological interactions among cytokines.

Wu et al. [14] attributed weakening of cytokine correlations in the glioma microenvironment to a transformation from a microenvironment controlled regulatory mechanism to a tumor controlled mechanism. Whether this transformation is reflected in the diminution of peripheral cytokine signaling in future glioma patients is unknown. In a study using a mouse model, Kennedy et al. [36] found that the immunomodulatory effects of the tumor on the periphery preceded those in the central nervous system. Therefore, the weakening of cytokine correlations that we observed may reflect changes in immune function characteristic of the prediagnostic periphery rather than those in the tumor microenvironment.

After glioma diagnosis, previous literature indicates that circulating cytokine levels and tumor cytokine expression are comparable [17]. For example, in glioblastoma patients, levels of the immunosuppressive cytokines IL4 and IL10 were elevated in both their peripheral lymphocytes and cell cultures from their tumors [37]. Studies of the 12 cytokines shown in the heat maps in Figs 2 and 3 find that glioma patients have higher levels of these cytokines than do their controls [16, 17]. Thus, for these 12 cytokines, only our results for VEGF are consistent with the post diagnostic literature [16, 17, 26]. Differences between our prediagnostic results and post diagnostic findings may be attributed, in part, to progressive tumor growth accompanied by increases in tumor-induced immunomodulation [10] or the effects of medication [38] and treatment [39]. Immunomodulation associated with tumor growth is complex and involves many levels of interaction [10]. Thus the primary contribution of the present work is that it further describes previously unknown prediagnostic cytokine changes. (For additional references on circulating cytokines in glioma patients see the S6 Table).

The primary limitation of the present study is that we do not have serial values for each study participant so that we cannot determine the within-person effects of time to diagnosis on peripheral cytokine levels and correlations. However, except for those within five years of diagnosis, control correlation matrices are similar, within levels of time before diagnosis, suggesting that our findings are not attributable to differences among study participants over time. Another potential limitation is that the sample in which we observe the weakening of correlations is relatively small (n = 55 cases, 55 controls). Again, we suggest that the similarity of control correlation patterns within five years before diagnosis to those further from the time of diagnosis indicates that our results may not be exclusively attributable to sampling variation. In addition, the preponderance of odds ratios less than one in this time category suggests that patterns observed in the heat maps within five years before diagnosis suggesting diminished cytokine signaling.

Our correlation findings should be interpreted in the context of the increased understanding of the two-way interaction between the central nervous system and the peripheral immune system [6, 40]. Specifically, the recent discovery of a lymphatic system in the meninges which is able to transport immune cells to the cervical lymph nodes provides further evidence of communication between the brain and the peripheral nervous system [41]. Although our findings can be placed in the context of brain- peripheral immune system communication, the specific details of this process, as they relate to gliomagenesis, are yet to be determined.

We have identified five serum cytokines and a cytokine-soluble receptor interaction each associated with changes in the preclinical risk of glioma. In addition, our findings of weakening case cytokine correlations within five years before diagnosis are consistent with those in our previous study of allergy-related cytokines [1818]. Further studies are needed to attempt replication of our results in different populations. Assuming they can be replicated, preclinical studies of additional immune function biomarkers, including immune function cells, should be conducted with the ultimate goal of identifying signs of gliomagenesis in its earliest stages.

## Supporting information

S1 Fig *P*-values from tests of correlations among cytokines ≤ 5 years before diagnosis. (DOCX)

S2 Fig Correlations among cytokines ≤ 5 years before diagnosis of glioblastoma and other glioma. (TIF)

S3 Fig Correlations among all 277 cytokines. (DOCX)

S1 Table Descriptive characteristics of glioma (grades 1–3) and glioblastoma (grade 4). (DOCX)

S2 Table Estimated odds ratios for all 277 cytokines by time before diagnosis. (XLSX)

S3 Table Case and control means and 95% confidence intervals of standardized logs of serum cytokine levels (within five years before diagnosis). (DOCX)

S4 Table Case and control means and 95% confidence intervals of standardized logs of serum cytokine levels (more than 15 years before diagnosis). (DOCX)

S5 Table Median coefficients of variation (CV) by serum cytokine based on replicate values collected in different batches. (DOCX)

S6 Table Previous studies of case-control cytokine levels in Figs 2 and 3 afterglioma diagnosis. (DOCX)
