# Supplementary material for: A nested case-control study of 277 prediagnostic serum cytokines and glioma
Source: PLoS One. 2017 Jun 8;12(6):e0178705. doi: 10.1371/journal.pone.0178705 (PMC5464586; doi:10.1371/journal.pone.0178705)
Supplement: S1 Fig — (DOCX) [file pone.0178705.s001.docx]

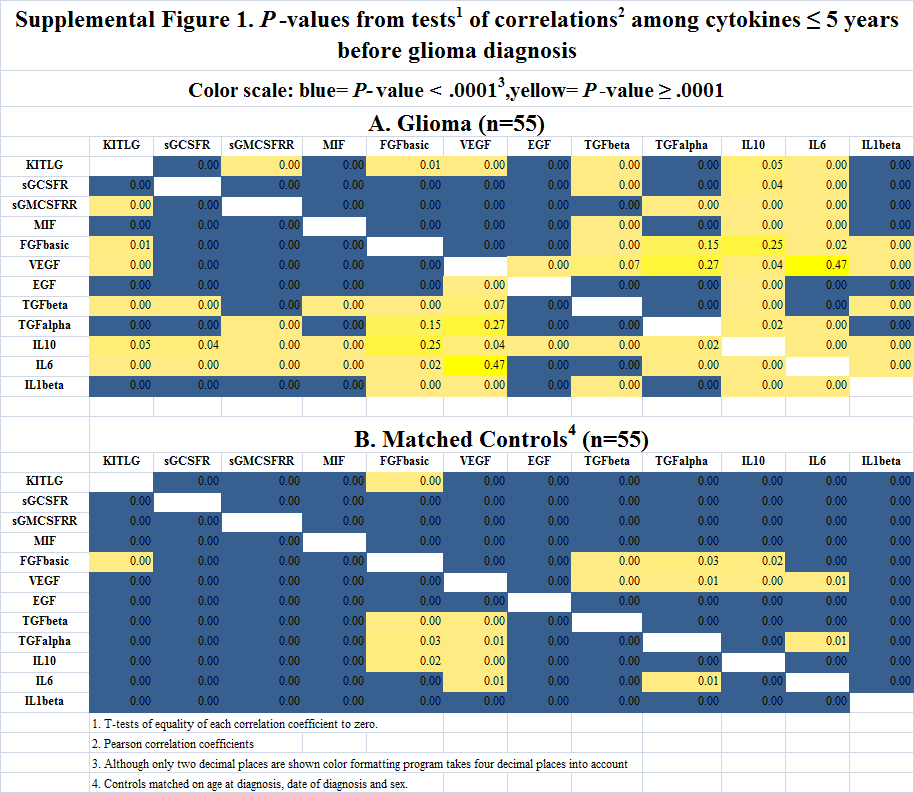


1. T-tests of equality of each correlation coefficient to zero
2. Pearson correlation coefficients
3. Although only two decimal places are shown color formatting program takes four into account
4. Controls matched on age and date of blood draw and gender.
5. For abbreviations see Fig. 2
