# Supplementary figures and images for: A nested case-control study of 277 prediagnostic serum cytokines and glioma
Source: PLoS One. 2017 Jun 8;12(6):e0178705. doi: 10.1371/journal.pone.0178705 (PMC5464586; doi:10.1371/journal.pone.0178705)

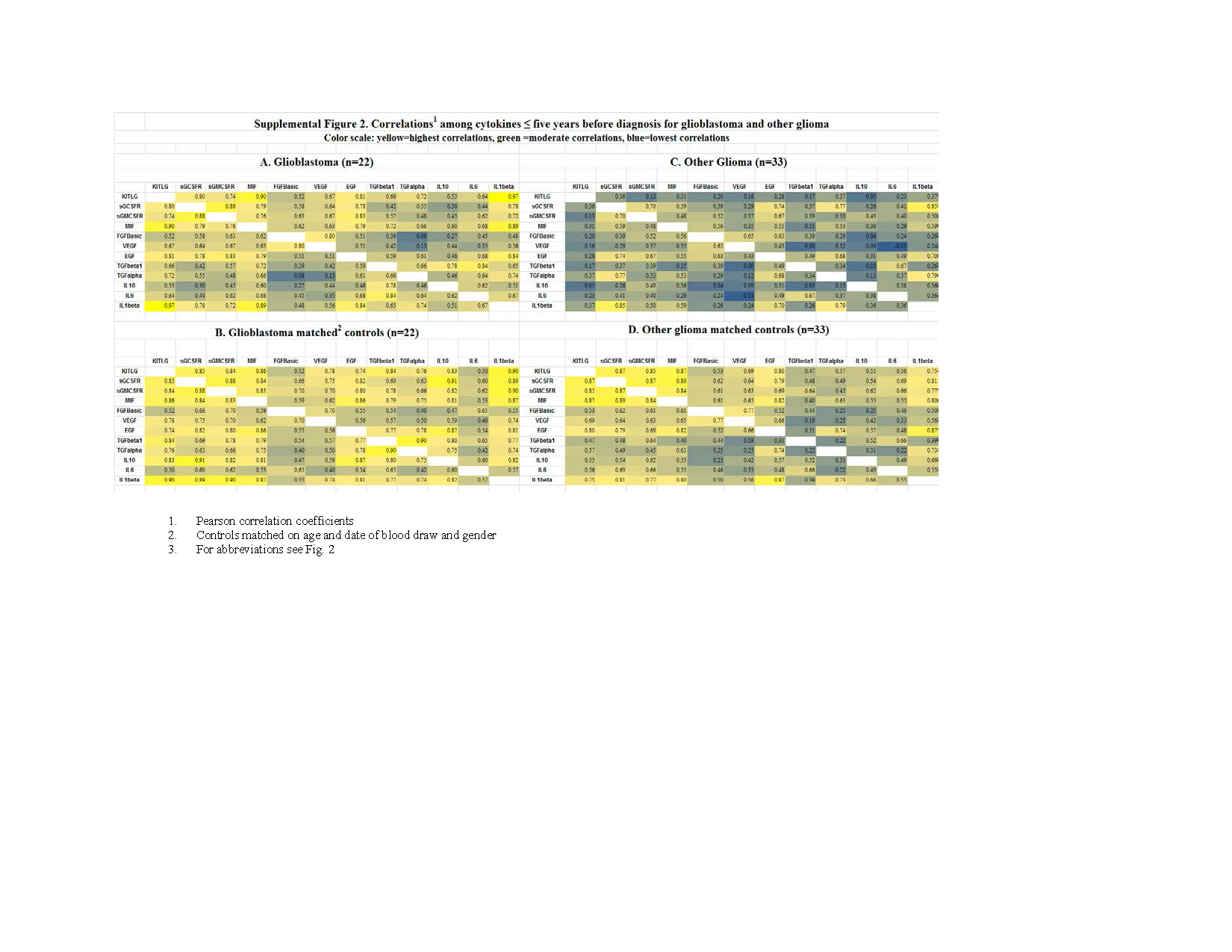

Supplement: S2 Fig — (TIF) [file pone.0178705.s002.tif]
