# Supplementary material for: A nested case-control study of 277 prediagnostic serum cytokines and glioma
Source: PLoS One. 2017 Jun 8;12(6):e0178705. doi: 10.1371/journal.pone.0178705 (PMC5464586; doi:10.1371/journal.pone.0178705)
Supplement: S3 Fig — (DOCX) [file pone.0178705.s003.docx]

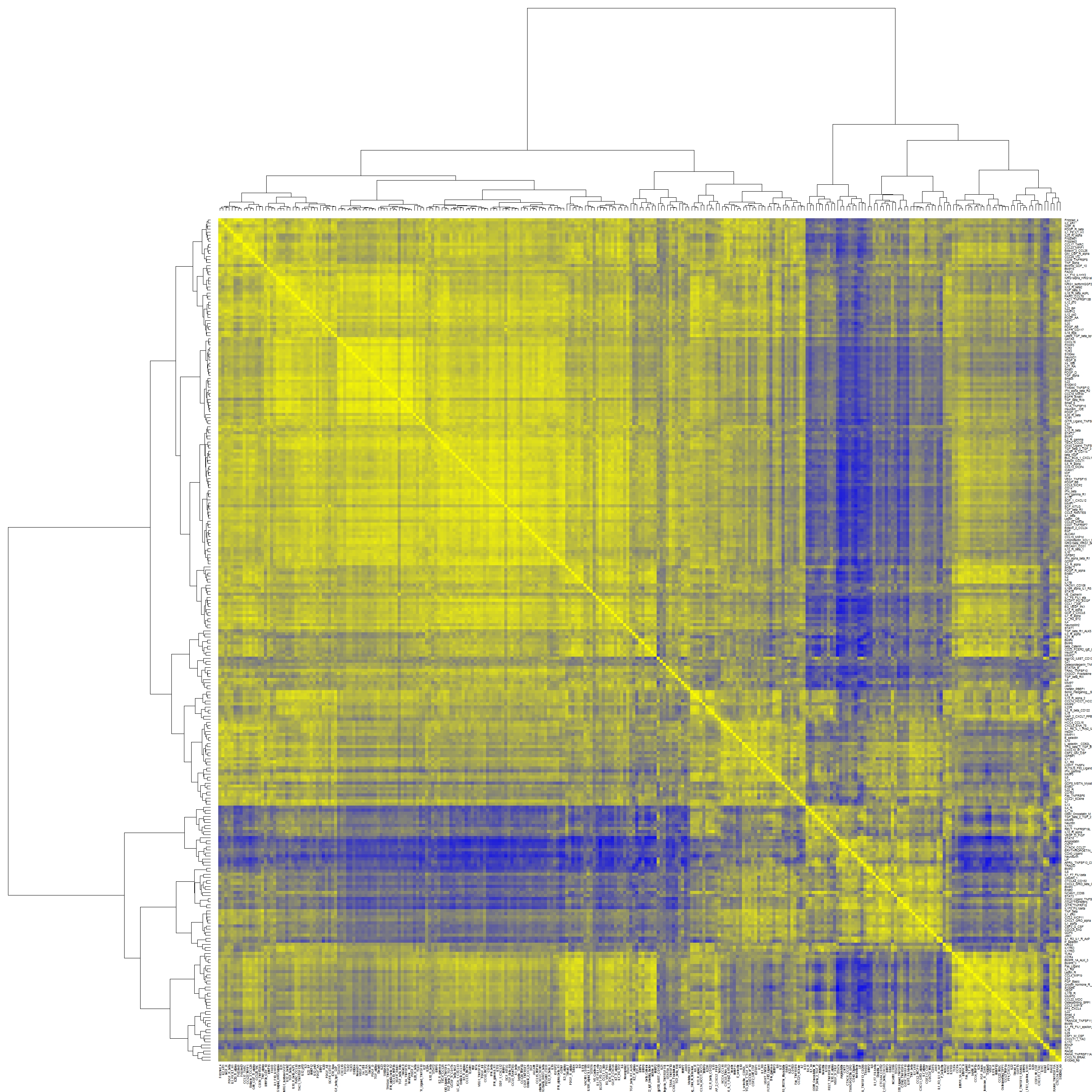


1. **Cases ≤ 5 years before diagnosis (yellow=high, blue=low).**

**Figure S3. Correlations among all 277 cytokines.**


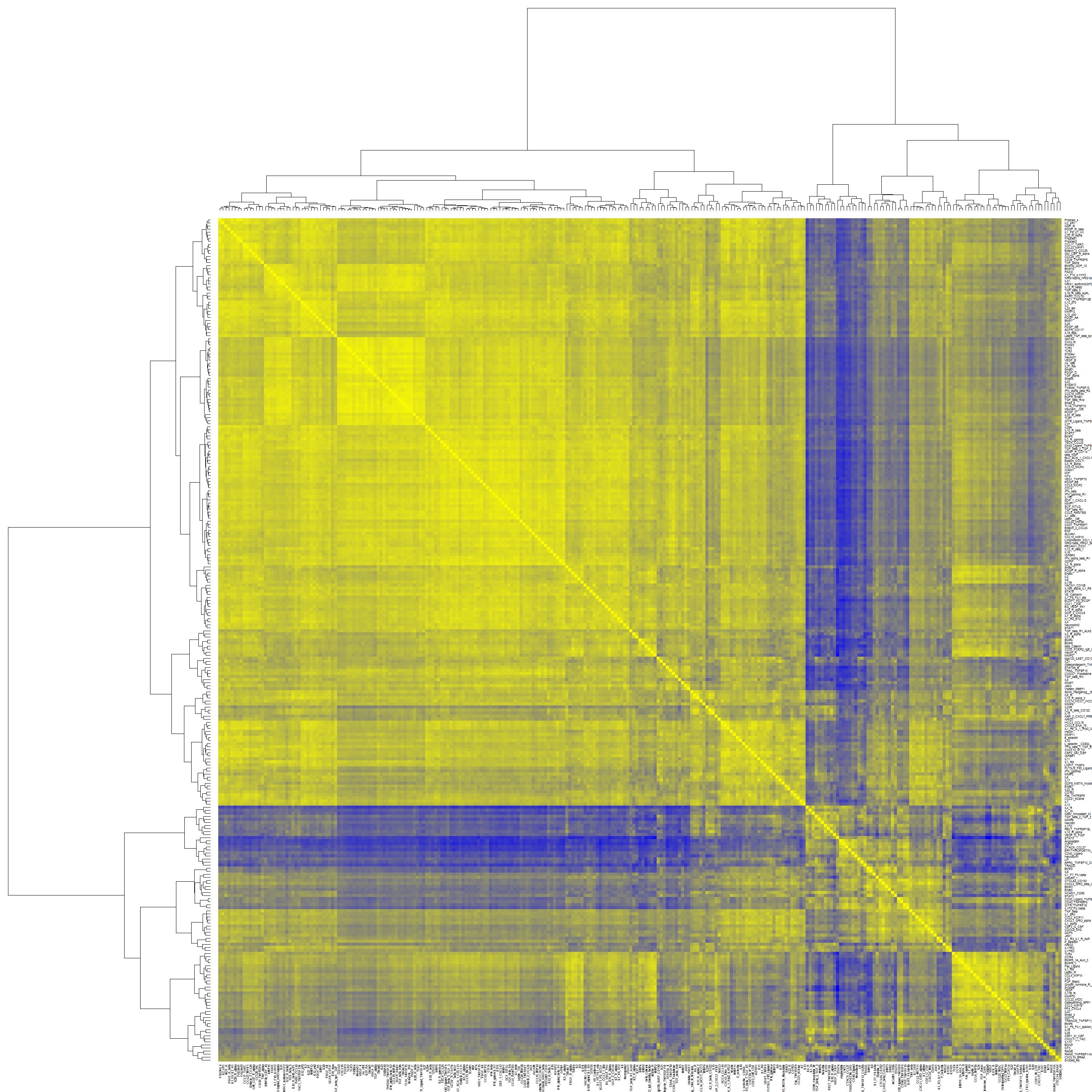


1. **Controls ≤ 5 years before diagnosis (yellow=high, blue=low).**

**Figure S3. Correlations among all 277 cytokines.**


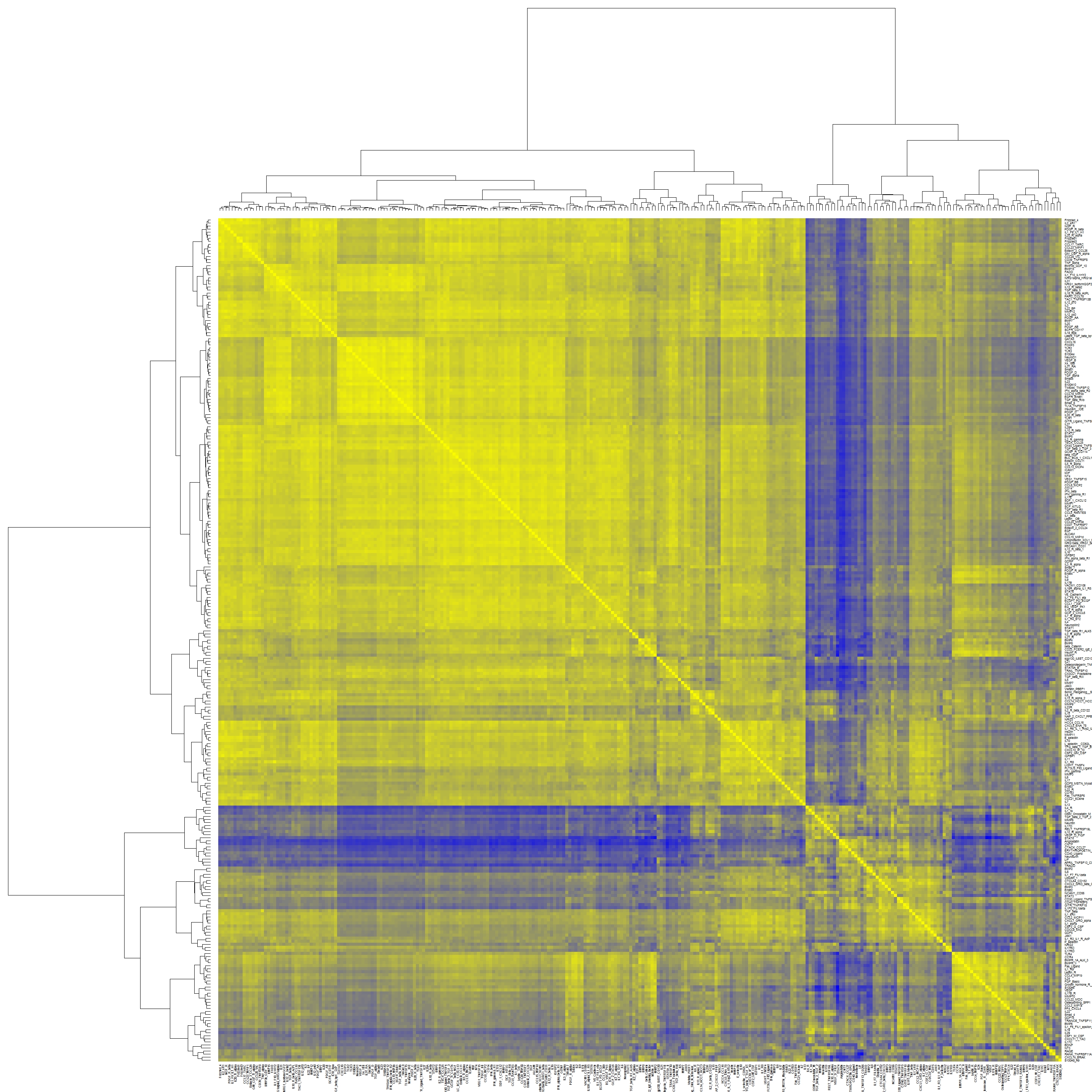


1. **Cases > 10 years before diagnosis (yellow=high, blue=low).**

**Figure S3. Correlations among all 277 cytokines.**


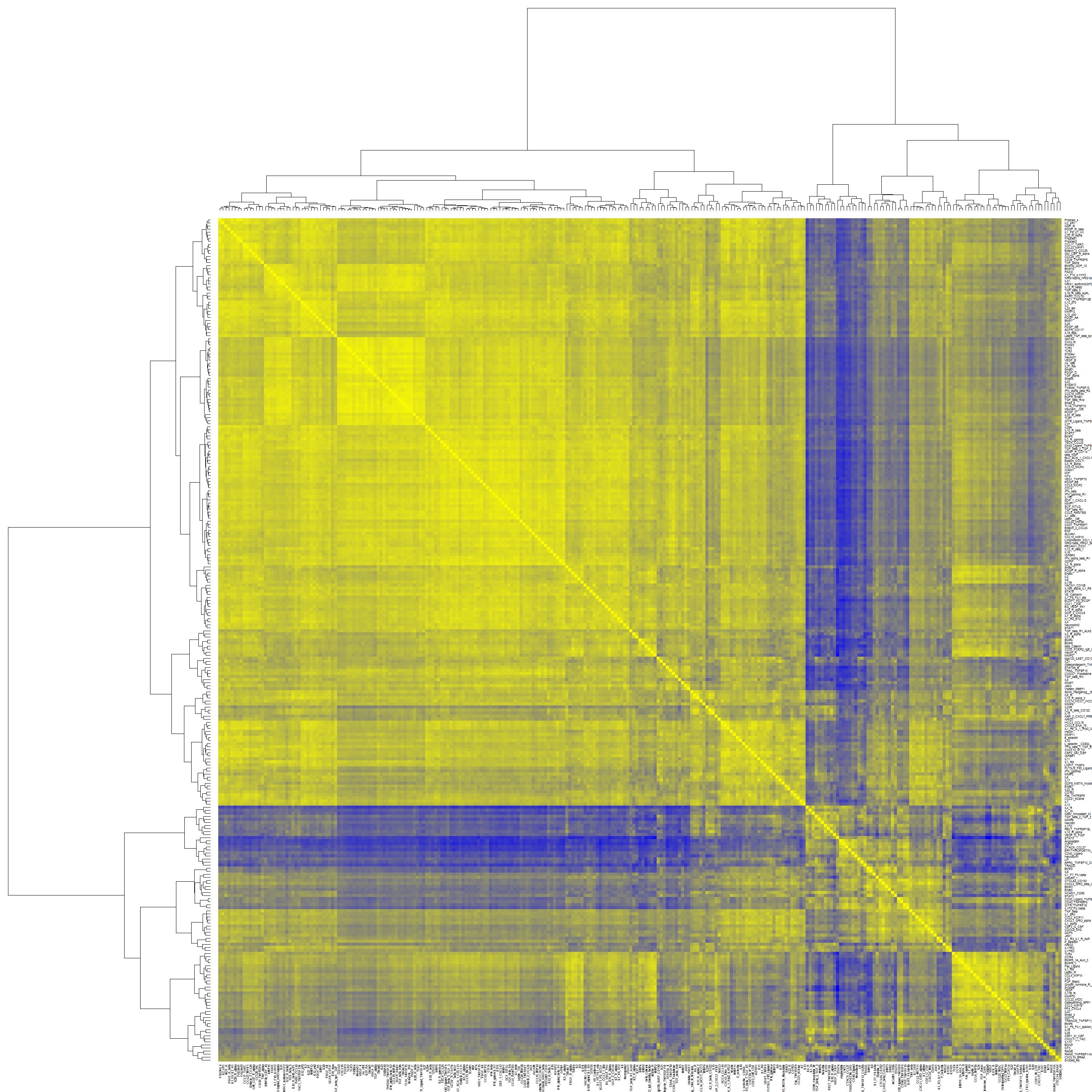


1. **Controls > 10 years before diagnosis (yellow=high, blue=low).**

**Figure S3. Correlations among all 277 cytokines.**
