# Supplementary material for: A nested case-control study of 277 prediagnostic serum cytokines and glioma
Source: PLoS One. 2017 Jun 8;12(6):e0178705. doi: 10.1371/journal.pone.0178705 (PMC5464586; doi:10.1371/journal.pone.0178705)
Supplement: S1 Table — (DOCX) [file pone.0178705.s004.docx]

**Supplemental Table 1 Descriptive characteristics of glioma (Grades 1-3) and glioblastoma (Grade 4)**

| **Descriptive Variables** | **Glioma**  **(Grades 1-3)** | **Controls** | **Glioblastoma**  **(Grade 4)** | **Controls ^2^** |
| --- | --- | --- | --- | --- |
| **All times before diagnosis** | | | | |
| Number | 172 | 172 | 315 | 315 |
| Percent men | 58 (51,66) | 58 (51, 66) | 72 (67, 77)^3^ | 72 (67, 77) |
| Median age at blood collection | 41 (40, 43) | 41 (40, 42) | 43 (42, 43)^4^ | 43 (42,43) |
| Median year of blood collection | 87 (77, 89) | 87 (77, 89) | 82 ( 81, 83) | 82 (81, 83) |
| Median age at glioma diagnosis | 54 (48, 60) | --- | 59 (58, 60) | ---^5^ |
| Median years blood collection to diagnosis | 12 (7, 17) | --- | 17 (16, 17) | --- |
| **≤ 5 years before diagnosis** | | | | |
| Number | 33 | 33 | 22 | 22 |
| Percent men | 48 (30, 66) | 48 (30,66) | 73 (53, 93)^3^ | 73 (53, 93) |
| Median age at blood collection | 41 (41,45) | 41 (41, 44) | 42 (41, 46)^4^ | 42 (41,47) |
| Median year of blood collection | 89 (86, 90) | 89 (86, 90) | 86 ( 73, 89) | 86 (74, 89) |
| Median age at glioma diagnosis | 45 (43, 47) | --- | 45 (44, 50) | ---^5^ |
| Median years blood collection to diagnosis | 3 (1,4) | --- | 3(2, 4) | --- |
| **> 10 years before diagnosis** | | | | |
| Number | 105 | 104 | 242 | 243 |
| Percent men | 47 (66, 100) | 46 (65, 100) | 67 (78, 100) | 67 (78, 100) |
| Median age at blood collection | 41 (40, 43) | 41 (40, 42) | 42 (40, 44) | 42 (40, 44) |
| Median year of blood collection | 86 (76, 89) | 86 (76, 89) | 82 (75, 88) | 82 (75, 89) |
| Median age at glioma diagnosis | 58 (54, 62) | --- | 60 (56, 67) | --- |
| Median years blood collection to diagnosis | 16 (14, 21) | --- | 18 (14, 24) | --- |
| **> 15 years before diagnosis** | | | | |
| Number | 61 | 61 | 167 | 169^6^ |
| Percent men | 51 (38, 64) | 51 38, 64) | 65 (59, 71) | 65 (59, 71) |
| Median age at blood collection | 41 (37, 43) | 41 (36, 42) | 42 (40, 44) | 42 (40, 44) |
| Median year of blood collection | 82 (76, 87) | 82 (76, 87) | 77 (75, 87) | 77 (1975, 1987) |
| Median age at glioma diagnosis | 61 (57, 65) | --- | 64 (59, 69) | --- |
| Median years blood collection to diagnosis | 21 (16, 26) | --- | 21 (18, 28) | --- |
| **> 20 years before diagnosis** | | | | |
| Number | 31 | 30 | 95 | 96 |
| Percent men | 55(36,73) | 57 (38,75) | 72 (62, 81) | 71 (62, 80) |
| Median age at blood collection | 39 (32, 43) | 39 (32, 42) | 42 (40, 45) | 42 (40, 44) |
| Median year of blood collection | 76 (74, 77) | 76 (74, 77) | 75 (74, 77) | 76 (73, 77) |
| Median age at glioma diagnosis | 65 (62, 68) | --- | 68 (64, 72) | --- |
| Median years blood collection to diagnosis | 26 (22, 31) | --- | 26 (23, 30) | --- |

1**.** Glioma study participants were blood donors (1974-2007) to the Janus Serum Bank, Oslo, Norway, who were subsequently diagnosed with glioma.

2. Control participants were individually matched to case participants on one-year age intervals, date of blood collection and sex.

3. 95% confidence interval

4. Interquartile range

5. Not applicable

6. Controls are matched to cases within three months of blood collection. Therefore a matched pair may fall into separate years before diagnosis categories thus accounting for the unequal numbers of cases and controls in that category
