# Supplementary material for: A nested case-control study of 277 prediagnostic serum cytokines and glioma
Source: PLoS One. 2017 Jun 8;12(6):e0178705. doi: 10.1371/journal.pone.0178705 (PMC5464586; doi:10.1371/journal.pone.0178705)
Supplement: S3 Table — (DOCX) [file pone.0178705.s006.docx]

**Supplemental Table 3. Case and control means and 95% confidence intervals of standardized logs of serum cytokine levels (within 5 years before diagnosis).**

**Cases Controls**

| **Variable** | **N** | **Mean** | **Lower 95% CL for Mean** | **Upper 95% CL for Mean** |
| --- | --- | --- | --- | --- |
| \| **KITLG** \| \| --- \| \| **sGCSFR** \| \| **sGMCSFR** \| \| **MIF** \| \| **FGFbasic** \| \| **VEGF** \| \| **EGF** \| \| **TGFbeta1** \| \| **TGFalpha** \| \| **IL10** \| \| **IL6** \| \| **IL1beta** \| | \| 55 \| \| --- \| \| 55 \| \| 55 \| \| 55 \| \| 55 \| \| 55 \| \| 55 \| \| 55 \| \| 55 \| \| 55 \| \| 55 \| \| 55 \| | \| -0.16 \| \| --- \| \| -0.06 \| \| -0.06 \| \| -0.11 \| \| 0.02 \| \| -0.02 \| \| -0.04 \| \| 0.04 \| \| -0.05 \| \| -0.04 \| \| -0.02 \| \| -0.12 \| | \| -0.45 \| \| --- \| \| -0.33 \| \| -0.34 \| \| -0.42 \| \| -0.28 \| \| -0.30 \| \| -0.33 \| \| -0.25 \| \| -0.34 \| \| -0.34 \| \| -0.29 \| \| -0.38 \| | \| 0.14 \| \| --- \| \| 0.22 \| \| 0.23 \| \| 0.19 \| \| 0.31 \| \| 0.25 \| \| 0.25 \| \| 0.33 \| \| 0.25 \| \| 0.26 \| \| 0.25 \| \| 0.14 \| |

| **Variable** | **N** | **Mean** | **Lower 95% CL for Mean** | **Upper 95% CL for Mean** |
| --- | --- | --- | --- | --- |
| \| **KITLG** \| \| --- \| \| **sGCSFR** \| \| **sGMCSFR** \| \| **MIF** \| \| **FGFbasic** \| \| **VEGF** \| \| **EGF** \| \| **TGFbeta1** \| \| **TGFalpha** \| \| **IL10** \| \| **IL6** \| \| **IL1beta** \| | \| 55 \| \| --- \| \| 55 \| \| 55 \| \| 55 \| \| 55 \| \| 55 \| \| 55 \| \| 55 \| \| 55 \| \| 55 \| \| 55 \| \| 55 \| | \| -0.05 \| \| --- \| \| -0.21 \| \| -0.22 \| \| -0.17 \| \| -0.04 \| \| -0.01 \| \| -0.23 \| \| -0.06 \| \| -0.08 \| \| -0.13 \| \| -0.15 \| \| -0.15 \| | \| -0.32 \| \| --- \| \| -0.44 \| \| -0.45 \| \| -0.39 \| \| -0.34 \| \| -0.30 \| \| -0.45 \| \| -0.29 \| \| -0.30 \| \| -0.30 \| \| -0.38 \| \| -0.36 \| | \| 0.23 \| \| --- \| \| 0.01 \| \| 0.01 \| \| 0.04 \| \| 0.26 \| \| 0.28 \| \| -0.00 \| \| 0.17 \| \| 0.14 \| \| 0.05 \| \| 0.08 \| \| 0.06 \| |
