# Supplementary material for: A nested case-control study of 277 prediagnostic serum cytokines and glioma
Source: PLoS One. 2017 Jun 8;12(6):e0178705. doi: 10.1371/journal.pone.0178705 (PMC5464586; doi:10.1371/journal.pone.0178705)
Supplement: S4 Table — (DOCX) [file pone.0178705.s007.docx]

| **Variable** | **N** | **Mean** | **Lower 95% CL for Mean** | **Upper 95% CL for Mean** |
| --- | --- | --- | --- | --- |
| \| **KITLG** \| \| --- \| \| **sGCSFR** \| \| **sGMCSFR** \| \| **MIF** \| \| **FGFbasic** \| \| **VEGF** \| \| **EGF** \| \| **TGFbeta1** \| \| **TGFalpha** \| \| **IL10** \| \| **IL6** \| \| **IL1beta** \| | \| 228 \| \| --- \| \| 228 \| \| 228 \| \| 228 \| \| 228 \| \| 228 \| \| 228 \| \| 228 \| \| 228 \| \| 228 \| \| 228 \| \| 228 \| | \| 0.11 \| \| --- \| \| 0.16 \| \| 0.11 \| \| 0.13 \| \| 0.01 \| \| 0.13 \| \| 0.11 \| \| 0.06 \| \| 0.06 \| \| 0.11 \| \| 0.06 \| \| 0.15 \| | \| -0.02 \| \| --- \| \| 0.01 \| \| -0.03 \| \| -0.01 \| \| -0.13 \| \| -0.00 \| \| -0.03 \| \| -0.07 \| \| -0.07 \| \| -0.03 \| \| -0.08 \| \| 0.01 \| | \| 0.25 \| \| --- \| \| 0.30 \| \| 0.25 \| \| 0.26 \| \| 0.16 \| \| 0.27 \| \| 0.24 \| \| 0.20 \| \| 0.18 \| \| 0.26 \| \| 0.19 \| \| 0.29 \| |

**Supplemental Table 4. Case and control means and 95% confidence intervals of standardized logs of serum cytokine levels (more than 15 years before diagnosis).**

**Cases Controls**

| **Variable** | **N** | **Mean** | **Lower 95% CL for Mean** | **Upper 95% CL for Mean** |
| --- | --- | --- | --- | --- |
| \| **KITLG** \| \| --- \| \| **sGCSFR** \| \| **sGMCSFR** \| \| **MIF** \| \| **FGFbasic** \| \| **VEGF** \| \| **EGF** \| \| **TGFbeta1** \| \| **TGFalpha** \| \| **IL10** \| \| **IL6** \| \| **IL1beta** \| | \| 230 \| \| --- \| \| 230 \| \| 230 \| \| 230 \| \| 230 \| \| 230 \| \| 230 \| \| 230 \| \| 230 \| \| 230 \| \| 230 \| \| 230 \| | \| 0.16 \| \| --- \| \| 0.16 \| \| 0.10 \| \| 0.17 \| \| -0.09 \| \| -0.03 \| \| 0.19 \| \| 0.09 \| \| 0.14 \| \| 0.14 \| \| 0.16 \| \| 0.16 \| | \| 0.02 \| \| --- \| \| 0.02 \| \| -0.04 \| \| 0.03 \| \| -0.21 \| \| -0.16 \| \| 0.05 \| \| -0.04 \| \| 0.00 \| \| 0.00 \| \| 0.02 \| \| 0.03 \| | \| 0.29 \| \| --- \| \| 0.29 \| \| 0.24 \| \| 0.30 \| \| 0.04 \| \| 0.09 \| \| 0.33 \| \| 0.23 \| \| 0.28 \| \| 0.28 \| \| 0.29 \| \| 0.29 \| |
