# Supplementary material for: A nested case-control study of 277 prediagnostic serum cytokines and glioma
Source: PLoS One. 2017 Jun 8;12(6):e0178705. doi: 10.1371/journal.pone.0178705 (PMC5464586; doi:10.1371/journal.pone.0178705)
Supplement: S5 Table — (DOCX) [file pone.0178705.s008.docx]

**Supplemental Table 5. Median coefficients of variation (CV) by serum cytokine^1^ based on replicate samples from different batches.**


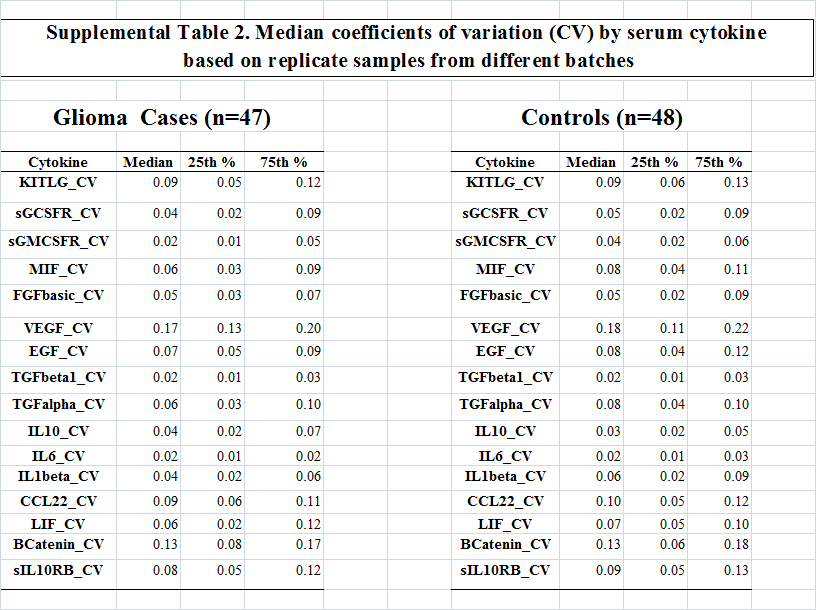


1. For abbreviations see Table 2 and Figs 2A and 2B.
