# Supplementary material for: A nested case-control study of 277 prediagnostic serum cytokines and glioma
Source: PLoS One. 2017 Jun 8;12(6):e0178705. doi: 10.1371/journal.pone.0178705 (PMC5464586; doi:10.1371/journal.pone.0178705)
Supplement: S6 Table — (DOCX) [file pone.0178705.s009.docx]

**Supplemental Table 6. Previous Studies of case-control cytokines in Figures 2 and 3 after glioma diagnosis ^a^.**

|  | Zhu[^1^](#_ENREF_1) | 18 serum cytokines[^2^](#_ENREF_2) | Review[^3^](#_ENREF_3) | Other |
| --- | --- | --- | --- | --- |
| KITLG^b^ |  |  |  | [^4^](#_ENREF_4) |
| sGCSFR |  |  |  | [^5^](#_ENREF_5) |
| sGMCSFR | *x* |  |  | [^6^](#_ENREF_6) |
| MIF |  |  | x | [^7^](#_ENREF_7) |
| FGFbasic |  | x |  |  |
| VEGF | x[^8^](#_ENREF_8) |  |  |  |
| EGF |  |  |  | [^9^](#_ENREF_9) |
| TGFB1 | x |  | x[^10^](#_ENREF_10) |  |
| TGFalpha |  |  |  | [^11^](#_ENREF_11) |
| IL10 | x[^12^](#_ENREF_12) | x | x | [^13^](#_ENREF_13)^,^[^14^](#_ENREF_14) |
| IL6 | x[^8^](#_ENREF_8)^,^[^12^](#_ENREF_12) | x | x |  |
| IL1beta |  |  |  | [^15^](#_ENREF_15) |

1. X in first three columns indicates cytokine is discussed in reference cited in column heading as well as in citations indicated by superscripts. Citations in last column come from sources indicated.
2. For abbreviations see Fig. 2

1 Zhu, V. F., Yang, J., Lebrun, D. G. & Li, M. Understanding the role of cytokines in Glioblastoma Multiforme pathogenesis. *Cancer Lett* **316**, 139-150, doi:10.1016/j.canlet.2011.11.001 (2012).

2 Nijaguna, M. B. *et al.* An Eighteen Serum Cytokine Signature for Discriminating Glioma from Normal Healthy Individuals. *PLoS ONE* **10**, e0137524, doi:10.1371/journal.pone.0137524 (2015).

3 Lippitz, B. E. Cytokine patterns in patients with cancer: a systematic review. *Lancet Oncol* **14**, e218-228, doi:10.1016/S1470-2045(12)70582-X (2013).

4 Sun, L. *et al.* Neuronal and glioma-derived stem cell factor induces angiogenesis within the brain. *Cancer Cell* **9**, 287-300 (2006).

5 Aliper, A. M., Frieden-Korovkina, V. P., Buzdin, A., Roumiantsev, S. A. & Zhavoronkov, A. A role for G-CSF and GM-CSF in nonmyeloid cancers. *Cancer medicine* **3**, 737-746, doi:10.1002/cam4.239 (2014).

6 Rafat, N., Beck, G., Schulte, J., Tuettenberg, J. & Vajkoczy, P. Circulating endothelial progenitor cells in malignant gliomas. *J Neurosurg* **112**, 43-49, doi:10.3171/2009.5.JNS081074 (2010).

7 Munaut, C., Boniver, J., Foidart, J. M. & Deprez, M. Macrophage migration inhibitory factor (MIF) expression in human glioblastomas correlates with vascular endothelial growth factor (VEGF) expression. *Neuropathol Appl Neurobiol* **28**, 452-460 (2002).

8 Reynes, G. *et al.* Circulating markers of angiogenesis, inflammation, and coagulation in patients with glioblastoma. *J Neurooncol* **102**, 35-41, doi:10.1007/s11060-010-0290-x (2011).

9 Selagea, L. *et al.* EGFR and C/EBP-b oncogenic signaling is bidirectional

in human glioma and varies with the C/EBP-b isoform. *FASEB J* (2016).

10 Hulshof, M. C., Sminia, P., Barten-Van Rijbroek, A. D. & Gonzalez Gonzalez, D. Prognostic value of plasma transforming growth factor-beta in patients with glioblastoma multiforme. *Oncol Rep* **8**, 1107-1110 (2001).

11 Brockmann, M. A. *et al.* Glioblastoma and cerebral microvascular endothelial cell migration in response to tumor-associated growth factors. *Neurosurgery* **52**, 1391-1399; discussion 1399 (2003).

12 Samaras, V. *et al.* Application of the ELISPOT method for comparative analysis of interleukin (IL)-6 and IL-10 secretion in peripheral blood of patients with astroglial tumors. *Mol Cell Biochem* **304**, 343-351 (2007).

13 Kumar, R. *et al.* Th1/Th2 cytokine imbalance in meningioma, anaplastic astrocytoma and glioblastoma multiforme patients. *Oncol Rep* **15**, 1513-1516 (2006).

14 Zisakis, A. *et al.* Comparative analysis of peripheral and localised cytokine secretion in glioblastoma patients. *Cytokine* (2007).

15 Nijaguna, M. B. *et al.* Definition of a serum marker panel for glioblastoma discrimination and identification of Interleukin 1beta in the microglial secretome as a novel mediator of endothelial cell survival induced by C-reactive protein. *J Proteomics* **128**, 251-261, doi:10.1016/j.jprot.2015.07.026 (2015).
